# Supplementary material for: Editorial for the Special -Issue on Biomechanics and Imaging
Source: Osteoarthr Imaging. 2025 Mar 20;5(1):100254. doi: 10.1016/j.ostima.2024.100254 (PMC13228666; doi:10.1016/j.ostima.2024.100254)
Supplement: Supplementary file 1 [file mmc1.docx]

**Editorial to the special issue on Biomechanics and Imaging**

Patrick Omoumi and Julien Favre

**Correspondence:**

Patrick Omoumi

Department of Radiology

Lausanne University Hospital and University of Lausanne

Bugnon 46

1011 Lausanne

Switzerland

This inaugural special issue of "Osteoarthritis Imaging" commemorates the 17^th^ International Workshop of Osteoarthritis Imaging (IWOAI), which we were honored to host in Lausanne, Switzerland, from June 28^th^ to 30^th^, 2023. This event centered on the interplay of imaging techniques and biomechanics in the context of osteoarthritis, highlighting significant advancements and promising perspectives in the field.

**Biomechanics and structure interact at the joint level**

Although the biomechanical and structural aspects of the joints have long been considered in the context of osteoarthritis, the importance of studying them concurrently has only recently been recognized. This combined approach is expected to contribute to a more thorough and comprehensive understanding.

For instance, the Integrated Joint System (IJS), a theoretical framework with emphasis on the relationships between biomechanical and structural features, has been proposed to model the initiation and progression of osteoarthritis [1]. To further enhance our understanding of this interplay, new data is needed, specifically focusing on features of the joint structure that relate with biomechanical features.

In this special issue of Osteoarthritis Imaging, we present a collection of 10 mini-reviews and 3 original research articles, by internationally recognized expert teams. These contributions offer valuable insights into imaging techniques that provide biomechanical and structural data, in the context of osteoarthritis.

**Quantifying joint biomechanics and structure using imaging**

Among the many biomechanical characteristics of the human joints, the kinetics and kinematics of the knee during walking have been shown to be particularly relevant to the development of osteoarthritis [2]. For example, there is a growing interest in studying gait in relation to tissue pathologies and morphology, as done in the IMI-APPROACH cohort [3].

The techniques available to measure the ambulatory biomechanics of the knee are also advancing rapidly. For example, significant efforts are being made to measure the movements of the bones using X-ray fluoroscopy [4]. This emergent approach allows to reduce measurement errors compared to traditional motion capture systems that use devices attached to the skin. Like for their traditional counterparts, the fluoroscopy systems can be associated with force sensors to quantify joint kinetics in addition to kinematics.

Developments toward more functional measures are occurring with cross-sectional imaging, as well. In particular, computed tomography (CT), which is traditionally acquired in a static fashion while the patients are lying down, has shown recent technological advances with the introduction of weight-bearing and movement as the joints are being imaged.

Weight-bearing CT has benefited from the introduction of the cone-beam technology, allowing for the imaging of joint structures as the patients are standing or moving in the scanner. This capability provides more mechanically relevant assessments of joint alignment and articular structures, particularly regarding the joint space width and the position of menisci [5, 6]. Furthermore, post-processing approaches have been developed to leverage the quantitative data which can be obtained that way [5].

Four-dimensional CT (4D CT) is another method that provides insights regarding joint function while the patient is lying down in the scanner [7]. 4D CT has been used to study normal and altered joint movement and explore the roles of soft tissues in various joints, including the knee.

Compared to CT, magnetic resonance imaging (MRI) offers greater contrast resolution, allowing for the assessment of soft tissues, including cartilage, synovium, menisci and ligaments. Research efforts have focused on leveraging these advantages and proposed methods to analyze knee structures during weight-bearing postures and movement. In particular, simulated weight-bearing and the use of open magnets have provided insight into meniscal biomechanics [8].

**Relationship between joint biomechanics and structure**

There has been a growing interest in imaging the articular structures that may be impacted in osteoarthritis in relation to their biomechanical environment. Bone, in particular, may undergo changes under the influence of increased mechanical loading, including increased bone formation leading to increased bone mineral density, geodes, and changes affecting its shape, such as bone attrition and the development of osteophytes. The tridimensional measurement of bone shape based on CT or MRI data, is an example of methods to quantify such changes[9]. Research has shown that bone shape can indicate the severity of osteoarthritis, predict its future onset, and correlate with clinical markers of osteoarthritis, including pain or total knee replacement (TKR). Therefore, bone shape may serve as a valuable imaging biomarker for osteoarthritis. This approach further benefits from recent advances in the fields of image segmentation, which facilitate access to such evaluations.

Sodium Fluoride positron emission tomography ([18F]NaF PET) is a new method that uses the dynamic nature of bone and its capacity to rapidly react to mechanical stress [10]. This imaging modality probes areas of newly formed bone and was shown in preclinical and clinical studies to be a quantitative tool to study the joint response to loading. This could show especially useful when integrated in biomechanical modeling of the joint to examine the interplay between bone metabolism and loading.

The relationship between biomechanics and the ultrastructure of cartilage, as assessed by compositional MRI techniques (particularly T2 and T1rho mapping), is another area of interest in the osteoarthritis imaging community [11]. For instance, studies have highlighted the effects of physical activity on cartilage, the impact of gait alterations after anterior cruciate ligament reconstruction on early degenerative disease, and the correlation between muscle strength, gait, and cartilage composition. In vitro studies have shown a correlation between indentation stiffness and cartilage composition [11].

**Global analysis and relationships modeling**

In order to leverage the extensive amount of information that could be acquired through 3D imaging in a meaningful manner, the data may be analyzed using standardized property maps [12]. These maps are particularly useful to study the spatial variations of tissue properties across the entire joint. The data from different modalities, each looking at a specific structural parameter, may be combined, and used to establish global models in relation to biomechanical data.

The advent of artificial intelligence will certainly help unlocking the complexity of joints and osteoarthritis modeling. For example, this technological progress enables more intricate analyses and simulations, leading to improved insights into joint biomechanics and structure, as well as into their interplay. This could enhance the identification of pathological changes in joint tissues indicative of osteoarthritis as well as the prediction of disease progression [13]. Progress also occurred in the field of computational biomechanics, where advanced models allow to integrate in vivo biomechanical data with structural data [14]. As an example, such model can inform on the muscle and joint contact forces.

In summary, the story of considering the joints globally is just unfolding. The methods reviewed in this special issue set the ground for further research studying large patient populations, and integrating clinical, biological, and genetic data to further enhance our understanding of osteoarthritis. Altogether, this is expected to enhance treatment, including through more personalized interventions. In the coming years, methodological enhancements are anticipated, especially in terms of improving accessibility.

As a final note, we would like to extend our gratitude to Frank Roemer for inviting us to compile this special edition. We also sincerely appreciate the contributions of all the authors who shared their insights and expertise. Collaborating on this issue has been a fascinating experience, and we are excited to share it with the scientific community.

REFERENCES

[1] S.N. Edd, P. Omoumi, T.P. Andriacchi, B.M. Jolles, J. Favre, Modeling knee osteoarthritis pathophysiology using an integrated joint system (IJS): a systematic review of relationships among cartilage thickness, gait mechanics, and subchondral bone mineral density. Osteoarthritis Cartilage. 26 (2018) 1425-37. https://doi.org/10.1016/j.joca.2018.06.017

[2] T.P. Andriacchi, J. Favre, The nature of in vivo mechanical signals that influence cartilage health and progression to knee osteoarthritis Current rheumatology reports. 16 (2014) 1-8. https://doi.org/10.1007/s11926-014-0463-2

[3] M.P. Jansen, D. Hodgins, S.C. Mastbergen, M. Kloppenburg, F.J. Blanco, I.K. Haugen, F. Berenbaum, F. Eckstein, F. Roemer, W. Wirth, Association between progression of knee osteoarthritis pathology and gait changes over two years: data from the IMI-APPROACH cohort Osteoarthritis …. (2024)

[4] N.B.J. Dur, M.G.H. Wesseling, E.M. Macri, J. Runhaar, Fluoroscopy: Taking a closer look at joint motion in osteoarthritis Osteoarthritis Imaging. (2024) https://doi.org/10.1016/j.ostima.2024.100240

[5] T.D. Turmezei, Quantitative assessment of the knee joint from weight bearing computed tomography Osteoarthritis Imaging. (2024) https://doi.org/10.1016/j.ostima.2024.100177

[6] S.E. Ghobrial, C.J. Tonkin, N.A. Segal, A.H. Gee, G.M. Treece, J.A. Lynch, M. Nevitt, K.E.S. Poole, F.W. Roemer, A. Guermazi, T.D. Turmezei, Weight bearing 3-D joint space width distribution at the knee varies according to location and extent of meniscal extrusion: a MOST investigation Osteoarthritis …. (2024)

[7] E. Ghotbi, H.A. Ibad, R. Hadidchi, F. Baffour, S. Demehri, A minireview of four-dimensional CT and joint biomechanics Osteoarthritis Imaging. (2024) https://doi.org/10.1016/j.ostima.2024.100241

[8] J.S. Broberg, D.R. Wilson, MR imaging methods to study meniscal position and mechanics Osteoarthritis Imaging. (2024) https://doi.org/10.1016/j.ostima.2024.100222

[9] A.D. Brett, P.G. Conaghan, 3-dimensional bone shape and knee osteoarthritis: What have we learned? Osteoarthritis Imaging. (2024) https://doi.org/10.1016/j.ostima.2024.100178

[10] A. Goyal, L. Watkins, O. Bruce, A. Gatti, F. Kogan, Metabolic bone imaging and its relationship with biomechanics Osteoarthritis Imaging. (2024) https://doi.org/10.1016/j.ostima.2024.100242

[11] T.M. Link, R.B. Souza, X. Li, Compositional MR imaging of cartilage and joint mechanics Osteoarthritis Imaging. (2024) https://doi.org/10.1016/j.ostima.2024.100183

[12] P. Margain, J. Favre, B.M. Jolles, P. Omoumi, Standardized maps – an emerging approach to leverage quantitative information in knee imaging Osteoarthritis Imaging. 4 (2024) 100251. https://doi.org/10.1016/j.ostima.2024.100251

[13] M.E. Mononen, M.J. Turunen, L. Stenroth, S. Saarakkala, M. Boesen, Biomechanical modeling and imaging for knee osteoarthritis–is there a role for AI? Osteoarthritis …. (2024)

[14] K. Yang, M.G. Pandy, Recent Developments in Computational Modelling of the Knee Osteoarthritis Imaging. (2024) https://doi.org/10.1016/j.ostima.2024.100244
